# High‐definition transcranial direct current stimulation of the occipital cortices induces polarity dependent effects within the brain regions serving attentional reorientation

**DOI:** 10.1002/hbm.25764

**Published:** 2022-01-07

**Authors:** Yasra Arif, Christine M. Embury, Rachel K. Spooner, Hannah J. Okelberry, Madelyn P. Willett, Jacob A. Eastman, Tony W. Wilson

**Affiliations:** ^1^ Institute for Human Neuroscience Boys Town National Research Hospital Omaha Nebraska USA; ^2^ College of Medicine University of Nebraska Medical Center Omaha Nebraska USA; ^3^ Department of Psychology University of Nebraska Omaha Nebraska USA

**Keywords:** alpha, gamma, magnetoencephalography, oscillations, Posner, validity

## Abstract

Numerous brain stimulation studies have targeted the posterior parietal cortex, a key hub of the attention network, to manipulate attentional reorientation. However, the impact of stimulating brain regions earlier in the pathway, including early visual regions, is poorly understood. In this study, 28 healthy adults underwent three high‐definition transcranial direct current stimulation (HD‐tDCS) visits (i.e., anodal, cathodal, and sham). During each visit, they completed 20 min of occipital HD‐tDCS and then a modified Posner task during magnetoencephalography (MEG). MEG data were transformed into the time‐frequency domain and significant oscillatory events were imaged using a beamformer. Oscillatory response amplitude values were extracted from peak voxels in the whole‐brain maps and were statistically compared. Behaviorally, we found that the participants responded slowly when attention reallocation was needed (i.e., the validity effect), irrespective of the stimulation condition. Our neural findings indicated that cathodal HD‐tDCS was associated with significantly reduced theta validity effects in the occipital cortices, as well as reduced alpha validity effects in the left occipital and parietal cortices relative to anodal HD‐tDCS. Additionally, anodal occipital stimulation significantly increased gamma amplitude in right occipital regions relative to cathodal and sham stimulation. Finally, we also found a negative correlation between the alpha validity effect and reaction time following anodal stimulation. Our findings suggest that HD‐tDCS of the occipital cortices has a polarity dependent impact on the multispectral neural oscillations serving attentional reorientation in healthy adults, and that such effects may reflect altered local GABA concentrations in the neural circuitry serving attentional reorientation.

## 1 INTRODUCTION

Goal‐directed attentional engagement, disengagement, and shift are essential elements of attention orientation/reorientation and are commonly quantified using the well‐established Posner cueing paradigm (Posner, 1980). During this task, a cue is presented and in some cases this cue predicts the spatial location of the target (i.e., valid trial) and in other cases it does not (i.e., invalid trial). It is during these invalid trials that participants must reallocate their neural resources to the previously unattended location in order to perform the task successfully. Such attentional shifting is ubiquitous in daily functioning and the associated neural correlates have been extensively studied in healthy adults (Corbetta, Patel, & Shulman, 2008; Posner, 2016; Proskovec, Heinrichs‐Graham, Wiesman, McDermott, & Wilson, 2018; Thiel, Zilles, & Fink, 2004; Vossel, Geng, & Fink, 2014), aging (Arif, Spooner, Wiesman, Embury et al., 2020; Daselaar, Huijbers, Eklund, Moscovitch, & Cabeza, 2013), and many other populations (Arif, Wiesman et al., 2020; Georgiou‐Karistianis, Churchyard, Chiu, & Bradshaw, 2002; Jimenez et al., 2016). Many of these studies have focused on neural activity in several nodes of dorsal and ventral attention networks (i.e., DAN and VAN), mainly frontal, parietal, and primary visual cortices.

Recently, emerging neuromodulatory techniques such as transcranial direct‐current stimulation (tDCS) has been used to target these regions, with the goal of altering the cortical activity governing attentional processing in a polarity‐dependent manner (Wiesman et al., 2018). Broadly speaking, tDCS is believed to modulate neural populations by altering resting membrane thresholds without triggering action potentials, and is conventionally conducted using sponge electrodes (Esmaeilpour et al., 2020; Jacobson, Koslowsky, & Lavidor, 2012; Jang et al., 2009; Lefaucheur & Wendling, 2019; Liebetanz, Nitsche, Tergau, & Paulus, 2002; Nitsche et al., 2003a; Stagg, Antal, & Nitsche, 2018). Further, anodal and cathodal stimulations are thought to enhance and diminish cortical excitability, possibly by inhibiting GABAergic and glutamatergic neurotransmission, respectively (Coffman, Clark, & Parasuraman, 2014; Fertonani & Miniussi, 2017; Filmer, Dux, & Mattingley, 2014; Nitsche et al., 2003b; Nitsche & Paulus, 2000, 2001). For instance, some prior studies have shown increased spontaneous alpha activity following anodal occipital stimulation during selective attention and visual processing (Heinrichs‐Graham, McDermott, Mills, Coolidge, & Wilson, 2017; McDermott et al., 2019; Wiesman et al., 2018; Wilson, McDermott, Mills, Coolidge, & Heinrichs‐Graham, 2018) and a decrease in spontaneous alpha and gamma power when stimulated with the opposite cathodal polarity (Marshall, Esterer, Herring, Bergmann, & Jensen, 2016; Wiesman et al., 2018). However, the findings of polarity‐dependent tDCS on multi‐spectral, task‐induced oscillations remain relatively inconsistent.

Importantly, several hypothetical models of how neuromodulation impacts brain function exist, and this is an area of major, active investigation (Jackson et al., 2016; Lafon, Rahman, Bikson, & Parra, 2017). The interest in the field is also fueled by the potential improvement seen in neurocognitive performance (e.g., attention) induced by non‐invasive interventions (Lo, van Donkelaar, & Chou, 2019), especially in the context of attentional redirection. For example, two previous studies targeting the posterior parietal cortex (PPC) showed enhanced attentional orienting/reorienting responses on an attention network test following anodal tDCS of PPC (Lo et al., 2019; Roy, Sparing, Fink, & Hesse, 2015). These findings were replicated in a study by Minamoto et al. (2014), who found that online anodal stimulation of PPC facilitated stimulus‐driven attentional processing in participants performing a reading span test. While these studies have provided critical data on how attentional processing and reallocation is affected by tDCS of the PPC, far less is known about how tDCS of earlier brain regions (e.g., visual cortices) affects attentional orienting. One previous study focused on selective attention and found that anodal occipital stimulation inhibited performance and modulated theta and alpha activity (McDermott et al., 2019). However, beyond this tDCS work, the quantification of altered population‐level cortical dynamics in early visual regions, as well as the downstream effects that stimulation in this region would have on higher regions serving attentional reorientation have yet to be determined. Notably, with the continuous advancement in tDCS methods, there has been an increase in the application of high‐definition tDCS (HD‐tDCS) (Arif, Spooner, Wiesman, Proskovec et al., 2020; Koshy et al., 2020; Spooner, Eastman, Rezich, & Wilson, 2020), which allows better focality and longer duration effects as compared to traditional sponge electrodes (Datta et al., 2009; Datta, Elwassif, Battaglia, & Bikson, 2008; Edwards et al., 2013; Kuo et al., 2013).

Herein, we utilized HD‐tDCS with an occipital electrode configuration, a modified Posner task, and magnetoencephalographic (MEG) imaging to investigate offline modulation of the neural oscillatory dynamics serving attention reorientation in a cohort of 28 healthy young adults. Based on previous literature targeting other brain regions and using related tasks, we hypothesized that anodal and cathodal stimulation montages applied to the occipital cortices would have polarity‐specific effects on the neural oscillatory dynamics underlying attentional reorientation in the theta, alpha, and gamma range in multiple regions of the visual attention network (McDermott et al., 2019; Reteig, Talsma, Van Schouwenburg, & Slagter, 2017; To, Eroh, Hart, & Vanneste, 2018).

## 2 METHODS

### 2.1 Participants

Twenty‐eight right‐handed adults (15 females) with a mean age of 24.91 years, *SD* = 3.37 (range: 20–34 years) were enrolled in this study. Exclusionary criteria included any medical illness affecting CNS function (e.g., HIV/AIDS, lupus), any neurological or psychiatric disorder, history of head trauma, current substance abuse, and the MEG laboratory’s standard exclusion criteria (e.g., ferromagnetic implants). All experimental procedures conformed to the standards set by the Declaration of Helsinki. The study protocol was approved by the University of Nebraska Medical Center’s (UNMC) Institutional Review Board (IRB). A full description of the study was given to all participants, followed by written informed consent, which adhered to the guidelines set forth by the UNMC IRB.

### 2.2 High‐definition transcranial direct stimulation

**FIGURE 1:**
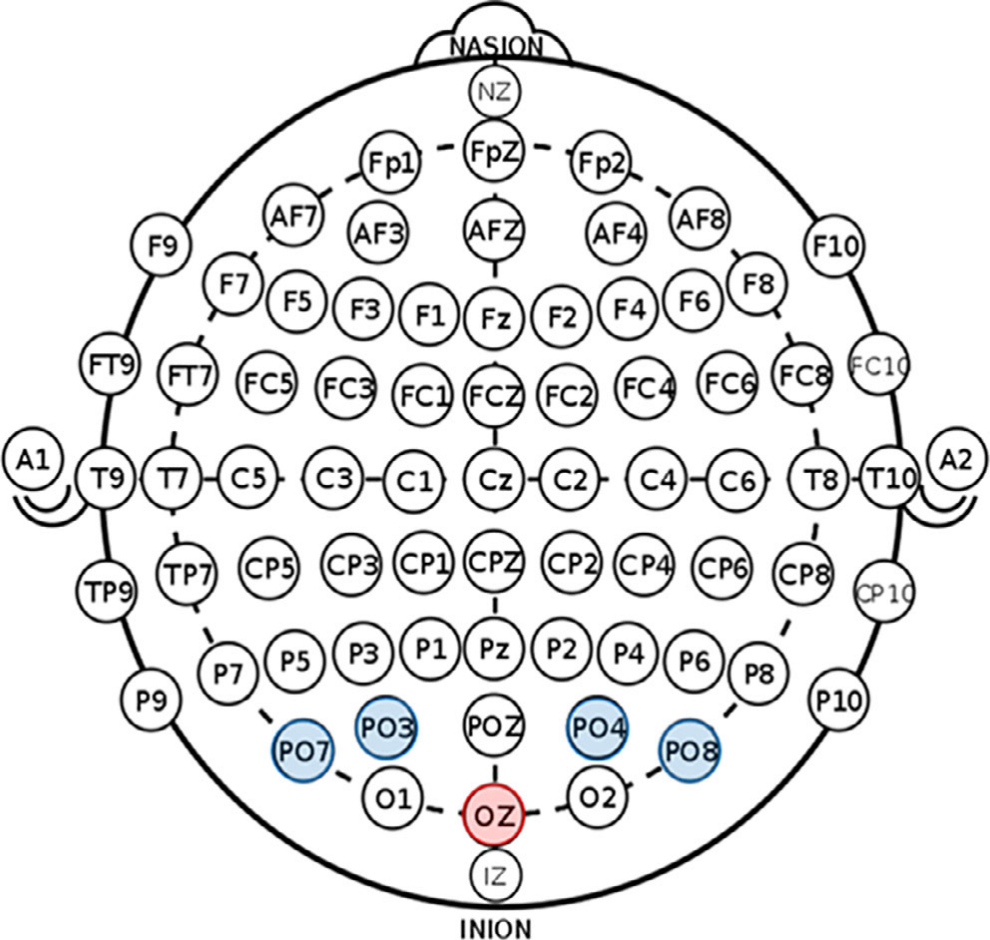
Two‐dimensional montage showing placement of HD‐tDCS electrodes. The central electrode was placed on Oz, which corresponds to the calcarine fissure based on an extension of the Okamoto et al. transformations of the scalp‐based international 10/20 system into MNI space and was surrounded on the superior and lateral sides by electrodes of opposite polarity near the parieto‐occipital junction (i.e., PO_3_, PO_4_, PO_7,_ and PO_8_)

**FIGURE 2:**
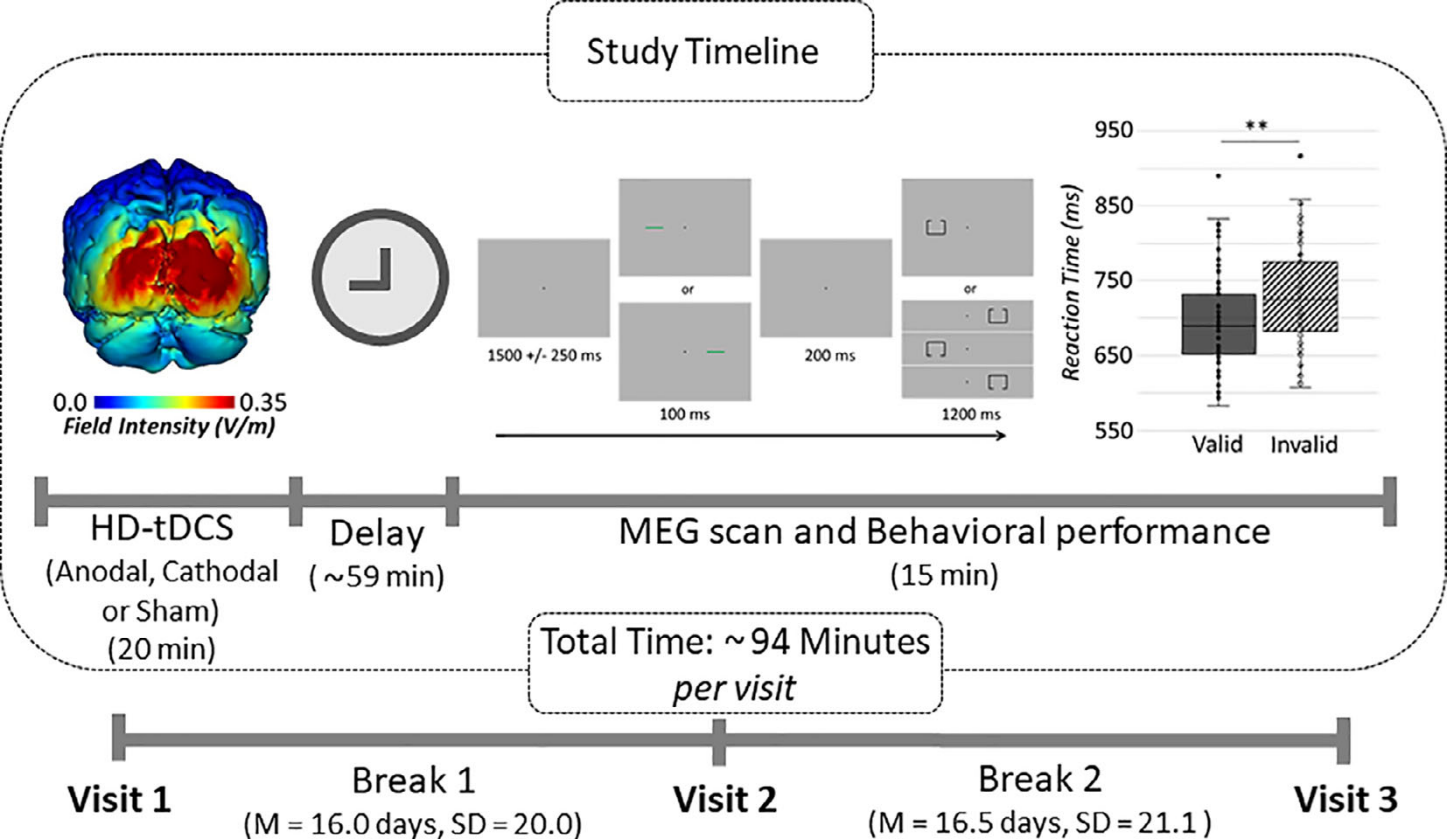
Study setup and behavioral performance: Participants received 20 min of anodal, cathodal, and sham HD‐tDCS over the occipital region (Oz). Stimulation conditions were pseudorandomized across three visits, each separated by at least 1 week. (Left) Current distribution modeling using our HD‐tDCS montage revealed focal stimulation of the occipital region. (Middle) About 1 hr after HD‐tDCS, participants completed a modified Posner paradigm during MEG recording. Briefly a fixation cross was presented for 1,500 ms (±250 ms), followed by a cue (green bar‐enhanced in the figure for better visualization) presented in the left or right visual hemifield for 100 ms. The target stimulus (box with opening) appeared 200 ms after cue offset in either the left or right visual hemifield, for 1,200 ms. Participants responded as to whether the opening was on the top or bottom of the target. The cue was presented on the same side as the target (i.e., valid condition) in half of the trials. A delay period was incorporated between HD‐tDCS and MEG recording to optimize offline effects; thus, the total time (i.e., from the beginning of stimulation to the end of the MEG task) took approximately 94 min. (Right) Performance of the task showed significant validity effects across all stimulation montages, such that participants responded more slowly to invalid compared to valid trials (*p < *.001)

A 4 × 1 configuration (a central electrode surrounded by four with opposite polarity; Soterix Medical; New York, NY) was utilized to deliver HD‐tDCS to the visual region (Oz), using the international 10/20 system (Jasper, 1958; Klem, Luders, Jasper, & Elger, 1999). Each electrode had a diameter of 12 mm and was comprised of Ag/AgCl. Cz was determined by the intersection of the inion/nasion plane and the preauricular plane following the procedures of the international 10/20 system. The central electrode was placed on Oz, which corresponds to the calcarine fissure based on an extension of the Okamoto et al. transformations of the scalp‐based international 10/20 system into MNI space (Okamoto et al., 2004; Okamoto & Dan, 2005), and was surrounded on the superior and lateral sides by electrodes of opposite polarity near the parieto‐occipital junction (i.e., PO_3_, PO_4_, PO_7,_ and PO_8_; Figure 1). This custom montage was designed to deliver maximum stimulation to early visual regions (i.e., V1–V4) within the occipital cortices, with minimal stimulation of neighboring parietal and temporal regions. Current density modeling indicated that this montage was largely successful in delivering current to the desired cortical regions (Figure 2).

Each participant completed three separate visits, at least a week apart (M = 16.25 days, *SD* = 20.5 days; Figure 2). Stimulation conditions were pseudorandomized to include one anodal, cathodal, and sham HD‐tDCS session. Participants were kept blind as to which visits encompassed active (anodal/cathodal) stimulations and sham. During the active visits, participants underwent 20 min of 2.0‐mA direct‐current stimulation, plus a 30‐s ramp‐up period, while in sham visits, no stimulation outside of the ramping procedure was applied. This approach was adopted to keep them unaware of which session they were in. Afterwards, participants were prepared for MEG recording and seated with their heads positioned within the MEG helmet. On averaged, it took about 59 min from the end of the stimulation to the start of the Posner task MEG recording, which fits well within the time span (i.e., 2 hr) considered appropriate to probe offline effects following stimulation (Kuo et al., 2013).

### 2.3 Experimental paradigm

The paradigm used in this study was a modified Posner task (Figure 2; Posner, 1980). During this task, the participants were seated in a magnetically shielded room and told to fixate on a crosshair presented centrally for 1,500 ms (± 250 ms). Following that, a green bar (the cue) was presented either to the left or right of the crosshair for 100 ms. The cue appeared on a given side 50% of all trials and could either be valid (presented on the same side as the upcoming target, 50% of all trials) or invalid (presented on the opposite side relative to the target). At 300 ms (200 ms after cue offset), a target was presented on either the left or the right side of the crosshair for 1,200 ms, and this was comprised of a box with an opening on either its top (50% of trials) or bottom. Participants were instructed to respond as to whether the opening was on the top (right middle finger) or the bottom (right index finger) of the box. Each target variant appeared an equal number of times, and each trial lasted 3,000 ms (± 250 ms). A total of 200 trials were used (100 valid, 100 invalid), leading to a total run‐time of approximately 14.5 min. Trials were pseudo‐randomly organized so that no more than three of the same target response or target/cue laterality pairs occurred in succession.

### 2.4 MEG data acquisition

All recordings were conducted in a one‐layer magnetically shielded room with active shielding engaged for environmental noise compensation. With an acquisition bandwidth of 0.1–330 Hz, neuromagnetic responses were sampled continuously at 1 kHz using an Elekta MEG system (Helsinki, Finland) with 306 sensors, including 204 planar gradiometers and 102 magnetometers. During data acquisition, participants were monitored via real‐time audio‐visual feeds from inside the shielded room. Each MEG dataset was individually corrected for head motion and subjected to noise reduction using the signal space separation method with a temporal extension (Taulu & Simola, 2006).

### 2.5 Structural MRI processing and MEG co‐registration

Prior to MEG measurement, four coils were attached to the subject’s head and localized, together with the three fiducial points and scalp surface, with a 3‐D digitizer (FASTRAK 3SF0002; Polhemus Navigator Sciences, Colchester, VT). Once the subjects were positioned for MEG recording, an electric current with a unique frequency label (e.g., 322 Hz) was fed to each of the coils. This induced a measurable magnetic field and allowed each coil to be localized in reference to the sensors throughout the recording session. As coil locations were also known with respect to head coordinates, all MEG measurements could be transformed into a common coordinate system. With this coordinate system, each participant’s MEG data were co‐registered with their T1‐weighted structural MRI prior to source space analysis using BESA MRI (Version 2.0). Structural T1‐weighted MRI images were acquired using a Siemens Skyra 3‐Tesla MRI scanner with a 32‐channel head coil and an MP‐RAGE sequence with the following parameters: TR = 2,400 ms; TE = 1.94 ms; flip angle = 8°; FOV = 256 mm; slice thickness = 1 mm (no gap); voxel size = 1 × 1 × 1 mm. These data were aligned parallel to the anterior and posterior commissures and transformed into standardized space. Following source analysis (i.e., beamforming), each subject’s functional MEG images were also transformed into standardized space using the transform that was previously applied to the structural MRI volume and spatially resampled.

### 2.6 MEG preprocessing, time‐frequency transformation, and sensor‐level statistics

Eye blinks and cardiac artifacts were removed from the data using signal space projection, which was accounted for during source reconstruction (Uusitalo & Ilmoniemi, 1997). The continuous magnetic time series was divided into epochs of 2,300 ms duration, with 0 ms defined as the onset of the cue and the baseline being the −600 to 0 ms window before cue onset. Given our task and epoch design, the target onset occurred at 300 ms. Epochs containing artifacts were removed based on a fixed threshold method, supplemented with visual inspection. In brief, for each individual, the distribution of amplitude and gradient values across all trials were computed, and those trials containing the highest amplitude and/or gradient values relative to the full distribution were rejected by selecting a threshold that excluded extreme values. Importantly, these thresholds were set individually for each participant, as inter‐individual differences in variables such as head size and proximity to the sensors strongly affect MEG signal amplitude (M = 888.69, *SD* = 189.54). On average, 89.39 valid and 89.76 invalid trials per participant remained after artifact rejection and were used in subsequent analyses. To ensure there were no systematic differences in the number of trials per participant, an analysis of variance (ANOVA) was computed, and this showed no significant main effect of condition (F = 1.88, *p* = .181), stimulation (F = 1.60, *p* = .211), or interaction effect (F = 0.53, *p* = .591).

Artifact‐free epochs were transformed into the time‐frequency domain using complex demodulation (Hoechstetter et al., 2004), and the resulting spectral power estimations per sensor were averaged over trials to generate time‐frequency plots of mean spectral density. These sensor‐level data were normalized per time‐frequency bin using the respective bin’s baseline power, which was calculated as the mean power during the −600 to 0 ms baseline period. The specific time‐frequency windows used for source reconstruction were determined by statistical analysis of the sensor‐level spectrograms across all participants using the entire array of 204 gradiometers. Briefly, each data point in the spectrogram was initially evaluated using a mass univariate approach based on the general linear model. To reduce the risk of false‐positive results while maintaining reasonable sensitivity, a two‐stage procedure was followed to control for Type 1 error. In the first stage, two‐tailed paired‐sample *t*‐tests against baseline were conducted on each data point, and the output spectrogram of *t*‐values was thresholded at *p* < .05 to define time‐frequency bins containing potentially significant oscillatory deviations across all participants. In stage two, time‐frequency bins that survived the threshold were clustered with temporally and/or spectrally neighboring bins that were also above the threshold (*p* < .05), and a cluster value was derived by summing the *t*‐values of all data points in the cluster. Nonparametric permutation testing was then used to derive a distribution of cluster values, and the significance level of the observed clusters (from Stage 1) was tested directly using this distribution (Ernst, 2004; Maris & Oostenveld, 2007). For each comparison, 10,000 permutations were computed. Based on these analyses, the time‐frequency windows that contained significant oscillatory events across all participants and conditions were subjected to the beamforming analysis.

### 2.7 MEG source imaging and statistics

Cortical neural responses were imaged using the dynamic imaging of coherent sources beamformer (Groß et al., 2001), which applies spatial filters in the time‐frequency domain to calculate voxel‐wise source power for the entire brain volume. The single images were derived from the cross‐spectral densities of all combinations of MEG gradiometers averaged over the time‐frequency range of interest and the solution of the forward problem for each location on a grid specified by input voxel space. Following convention, we computed noise‐normalized source power for each voxel per participant using active (i.e., task) and passive (i.e., baseline) periods of equal duration and bandwidth (Hillebrand, Singh, Holliday, Furlong, & Barnes, 2005) at a resolution of 4.0 × 4.0 × 4.0 mm. Such images are typically referred to as pseudo‐t maps, with units (pseudo‐t) that reflect noise‐normalized power differences (i.e., active vs. passive) per voxel. MEG pre‐processing and imaging used the Brain Electrical Source Analysis (version 6.1) software.

After imaging, average whole‐brain maps were computed across all conditions (valid and invalid), stimulations (anodal, cathodal, and sham), and participants for the selected time‐frequency windows. These 3D maps of brain activity were used to assess the anatomical basis of the significant oscillatory responses identified through the sensor‐level analysis. The maps were further used to identify the peak voxel (i.e., the voxel with the maximum amplitude value) per time‐frequency response. Finally, response amplitude values were extracted from these peak voxels and compared statistically using repeated measures 2 × 3 ANOVAs to identify differences between task conditions and stimulation conditions, as well as their interaction. All ANOVAs were tested for violations of sphericity using Mauchly’s test and where needed were corrected using the Greenhouse–Geisser correction. Follow‐up tests for conditional differences were conducted using traditional paired‐samples *t*‐tests. Any values ±3 *SD* from the mean was considered an outlier and removed prior to statistical analyses.

## 3 RESULTS

All participants successfully completed the study, but three were excluded from the behavioral analysis due to poor performance (i.e., reaction time greater or accuracy lesser than 3 *SD* from the mean). The remaining 25 participants (14 females) had a mean age of 25.09 years with *SD* = 3.37 (range: 20–34 years).

### 3.1 Behavioral effects

A repeated measures 2 × 3 ANOVA on the behavioral data showed a significant main effect of condition for reaction time, F(1,24) = 250.26, *p* < .001, such that participants responded more slowly during invalid trials (M = 730.98, *SD* = 66.76) relative to valid trials (M = 696.84, *SD* = 62.47; Figure 2), irrespective of the HD‐tDCS montage. This validity effect (i.e., invalid‐valid; Vossel, Thiel, & Fink, 2006) was 34.14 ms on average (*SD* = 17.45). Moreover, performance was near ceiling in both conditions and across all stimulation configurations (i.e., anodal: valid = 98.4%, invalid = 97.8%, cathodal: valid = 98.1%, invalid = 97.4%, sham: valid = 98.3%, invalid = 98.1%). Finally, no significant effect of HD‐tDCS montage was observed for reaction time, F(2,48) = 0.23, *p* = .74 or accuracy F(2,48) = 0.59, *p* = .56). Similarly, the condition by stimulation interactions were not significant for reaction time, F(2,48) = 0.62, *p* = .54 or accuracy, F(2,48) = 0.70, *p* = .50.

### 3.2 Sensor‐level analysis

**FIGURE 3:**
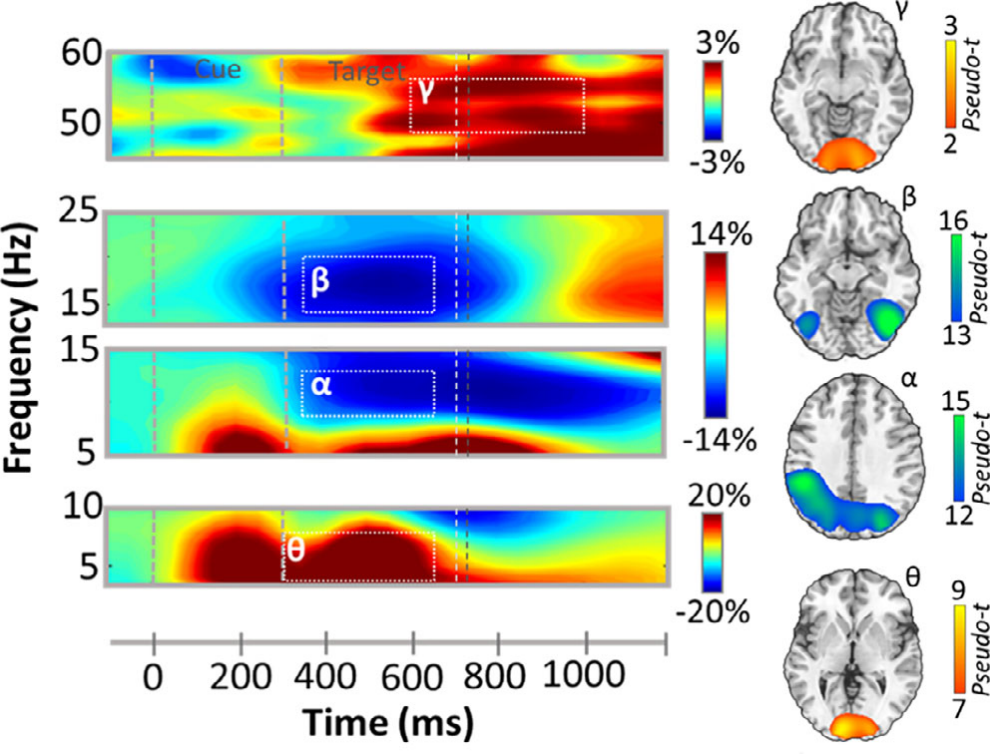
Neural responses to the modified Posner paradigm. (Left): Grand averaged time‐frequency spectrograms of MEG sensors exhibiting one or more significant responses, with gamma activity at the top, beta and alpha below, and theta at the bottom. In each spectrogram, time (ms) is denoted on the *x*‐axis and frequency (Hz) is shown on the *y*‐axis. All signal power data are expressed as percent difference from baseline, with color legends shown on the right of the spectrograms. Dashed rectangles indicate the time‐frequency windows that were subjected to beamforming. (Right): Grand‐averaged beamformer images (pseudo‐t) across all participants, conditions, and HD‐tDCS montages for each time‐frequency component, with theta at the bottom, alpha/beta in the middle, and gamma at the top. Separate color scale bars are shown for each

While strong neural responses were observed after cue onset, the present study aimed to investigate oscillations tied to the attentional reorienting process. Thus, our statistical analyses focused on neural responses during the target period (i.e., starting 300 ms after cue onset). These analyses revealed four spectrally specific oscillatory responses in gradiometers near the parietal, occipital, and frontal cortices across all participants, conditions, and stimulation montages (Figure 3). Briefly, during target presentation, a strong increase in the theta range (3–7 Hz) was observed from 300 to 650 ms (*p* < .001, corrected). This response partially overlapped in time with a robust desynchronization in the beta range (14–20 Hz; 350–650 ms, *p* < .001, corrected) and a slightly later decrease in the alpha range (8–13 Hz; 350–650 ms, *p* < .001, corrected). Finally, a strong gamma synchronization (49–57 Hz; 600–1,000 ms, *p* < .001, corrected) was observed, most prominently in sensors near the occipital cortices.

### 3.3 MEG beamformer imaging and statistics

To identify the spatial origin of these sensor‐level oscillatory responses, the above‐mentioned time‐frequency windows of interest were imaged using a beamformer, and the resulting maps per response were averaged over all participants, task conditions, and HD‐tDCS montages. Strong increases in theta amplitude were observed from 300 to 650 ms in bilateral occipital cortices and the left postcentral gyrus. In contrast, strong decreases in alpha activity were observed in lateral occipital cortices bilaterally, left superior parietal lobule, and the intraparietal sulcus, which ranged temporally from 350 to 650 ms. Likewise, strong decreases in beta activity were observed in bilateral occipital cortices and the left parietal cortices. Finally, the robust increase in gamma activity from 600 to 1,000 ms originated in the bilateral occipital cortices (Figure 3).

**FIGURE 4:**
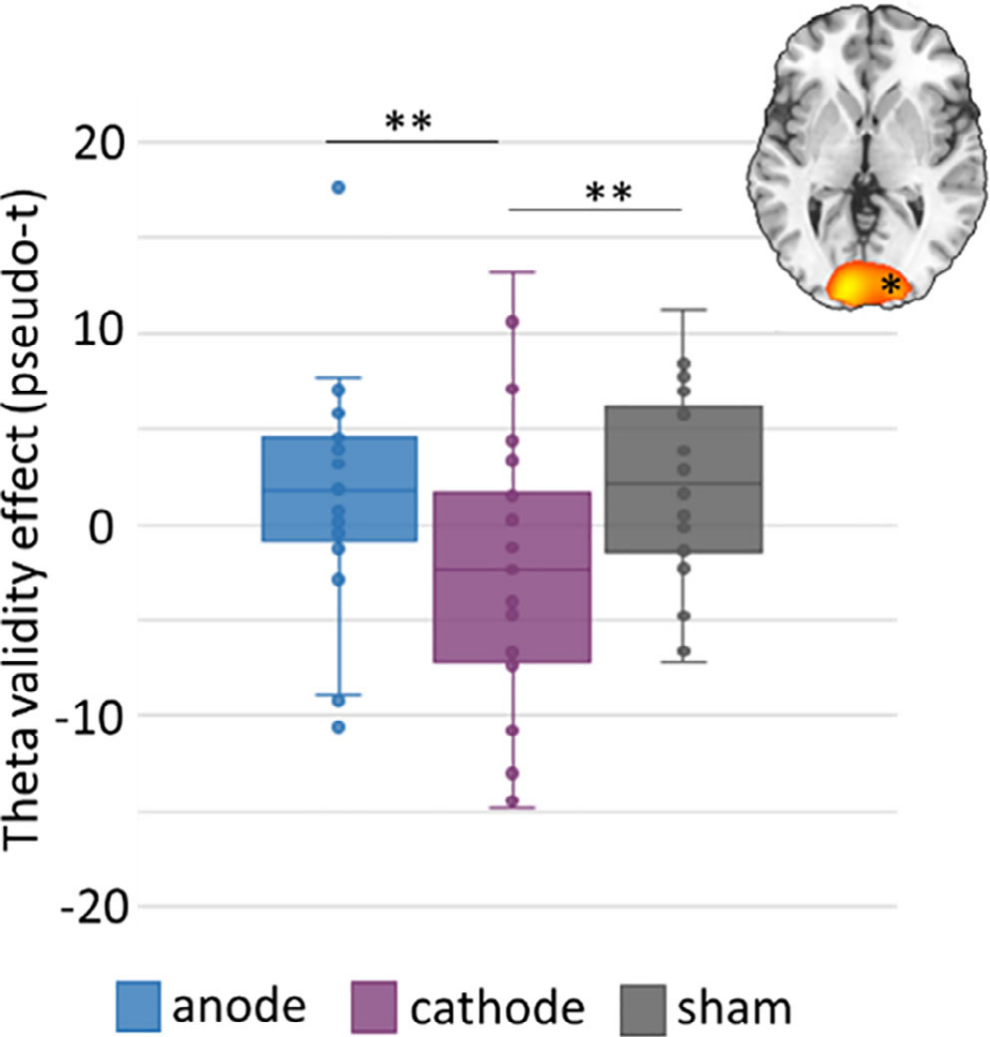
HD‐tDCS polarity dependent modulation of the theta validity effect during attentional reorientation. Response amplitude values (pseudo‐t) were extracted from peak voxels and subjected to a 2 × 3 ANOVA. A significant condition by montage interaction was found in the theta range in right occipital cortices, such that the theta validity effect was significantly weaker in this region following cathodal stimulation compared to both anodal and sham conditions. Box and whisker plot shows the right occipital theta validity in anodal, cathodal, and sham stimulation. Asterisk in the image to the upper right indicates the relevant region. *p < .05, ** *p* = .005

To determine the effects of task condition, stimulation montage, and their interaction, response amplitude values (pseudo‐t) were extracted from the peak voxels identified in these grand averaged maps and were compared statistically using repeated measures 2 × 3 ANOVAs. Two participants exhibited outlier theta responses and one had outlier alpha responses and these values were excluded from the final analyses. In regard to theta oscillations, the ANOVA revealed a significant stimulation montage by condition interaction effect in the right occipital cortices, F(2,52) = 6.03, *p* = .004, and post hoc paired sample *t*‐tests showed that the theta validity effect (invalid–valid trials) was significantly weaker following cathodal stimulation compared to both anodal, t(25) = 3.06, *p* = .005 and sham, t(25) = −3.10, *p* = .005 (Figure 4).

**FIGURE 5:**
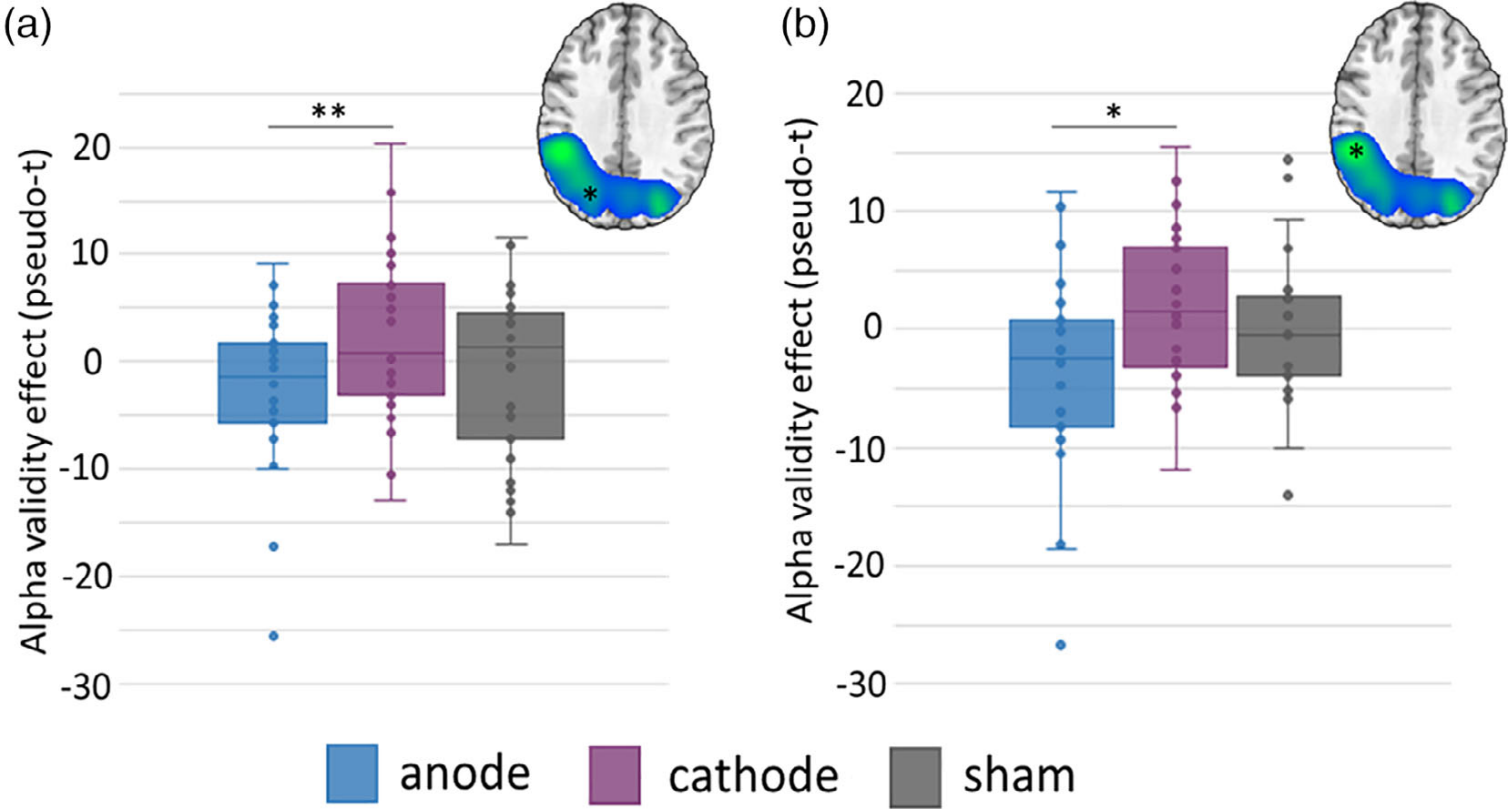
HD‐tDCS polarity dependent modulation of the alpha validity effect during attentional reorientation. Response amplitude values (pseudo‐t) were extracted from peak voxels and subjected to a 2 × 3 ANOVA. A significant stimulation montage by condition interaction was found in the alpha range and follow‐up testing indicated that the alpha validity effect was significantly weaker in the (a) left occipital and (b) left parietal following cathodal compared to anodal stimulation. Box and whisker plots show the individual data points, median (horizontal line), first and third quartile (box), and local minima and maxima (whiskers). Asterisks in the images above each plot indicate the relevant regions. *p < .05, ** *p* < .005

A similar task condition by stimulation montage interaction was observed for alpha range oscillations in the left occipital cortices, F(2,52) = 4.55, *p* = .015, and left superior parietal region extending inferior along the intraparietal sulcus, F(2,52) = 3.45, *p* = .039. Post hoc analysis showed that the alpha validity effect (invalid–valid trials) was weaker (i.e., less negative or even slightly positive) following cathodal compared to anodal stimulation in both left occipital, t(26) = −3.11, *p* = .005, and parietal cortices, t(26) = −2.23, *p* = .034 (Figure 5). No main effects or interactions were significant for beta frequency responses.

**FIGURE 6:**
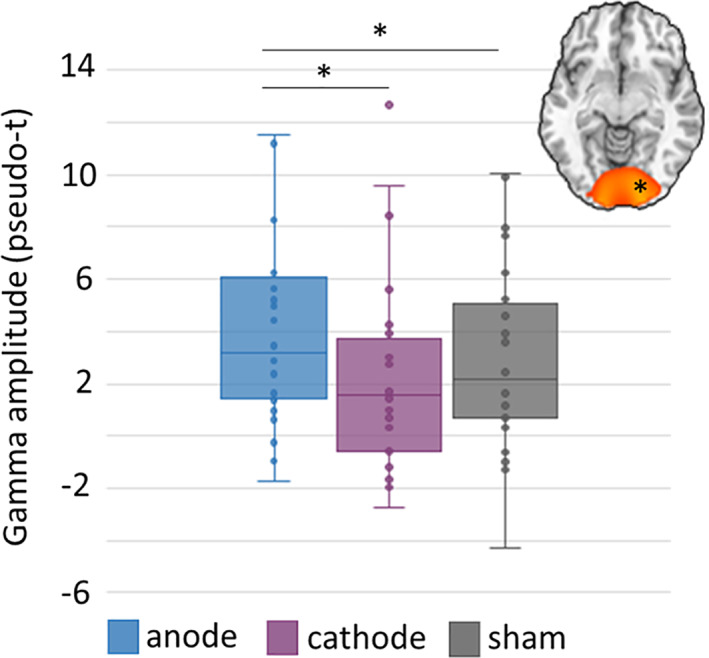
HD‐tDCS polarity dependent modulation of gamma oscillations during attentional reorientation. Response amplitude values (pseudo‐t) were extracted from peak voxels and subjected to a 2 × 3 ANOVA. A significant main effect of stimulation was found in the gamma range in the right occipital region, such that gamma oscillations were significantly stronger after anodal stimulation compared to the cathodal and sham conditions. Box and whisker plot shows right occipital gamma amplitude across both task conditions in anodal, cathodal, and sham conditions, and includes individual data points, median (horizontal line), first and third quartile (box), and local minima and maxima (whiskers). Asterisks in the image above each plot indicate the relevant region. *p < .05

In regard to gamma oscillations, we found a main effect of stimulation montage in the right occipital cortices, F(2,54) = 4.88, *p* = .018, and marginally in the left occipital (*p* = .07). Follow‐up paired sample *t*‐tests showed that participants exhibited increased gamma activity following anodal stimulation compared to both cathodal stimulation, cathodal t(27) = 2.58, *p* = .016, and sham t(27) = 2.25, *p* = .033, in the right occipital (Figure 6). The same pattern of results was observed in the left occipital. The main effect of task condition and the interaction were not significant.

**FIGURE 7:**
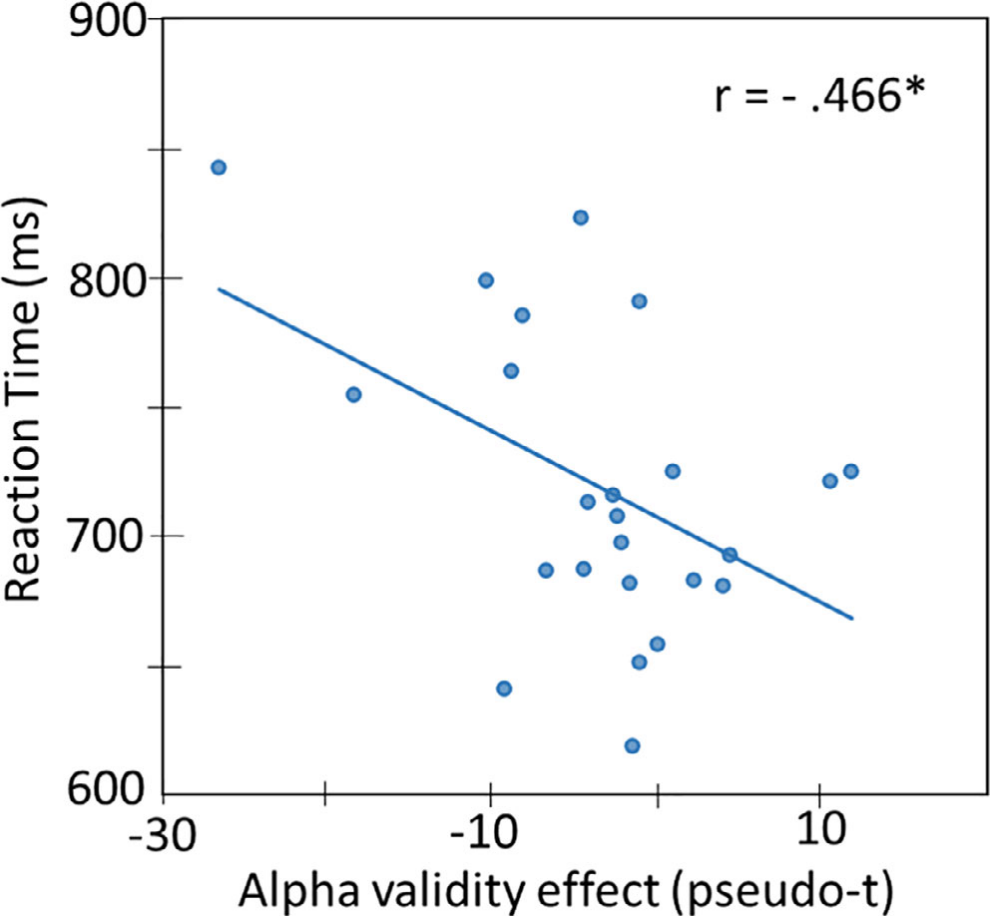
Correlation of alpha validity effect with reaction time. Pearson correlational analysis between the alpha validity effect in left parietal regions (*x*‐axis; pseudo‐t) and reaction time (*y*‐axis) following anodal stimulation showed a negative association (*p* < .05). The respective line of best fit and the *r* value are overlaid on the plot

Finally, to identify whether these neural effects were linked with behavioral metrics, a Pearson’s correlation was conducted between the amplitude of oscillations for the significant interaction effects noted above and reaction time. These analyses showed a significant negative association between the alpha validity effect in the left parietal and reaction time following anodal stimulation only, *r* = .466, *p* = .02 (Figure 7).

## 4 DISCUSSION

In the current study, we examined the polarity‐specific effects of HD‐tDCS of the occipital cortices on the offline multispectral neural oscillations sub‐serving attentional reorientation in a cohort of healthy adults. Our primary findings indicated that HD‐tDCS modulated neural validity effects in a polarity‐specific pattern in the theta range within the right occipital cortices, as well as alpha validity effects in the left occipital and parietal cortices. In addition, anodal stimulation of the occipital cortices significantly increased gamma oscillatory amplitude in right occipital regions across both task conditions. Finally, we observed a negative coupling between the strength of the alpha validity effect in the left parietal and reaction time, which was specific to anodal stimulation. Below we discuss the implications of these novel findings for understanding how polarity‐specific tDCS affects cortical oscillations during attentional reorientation.

The behavioral analysis indicated a significant validity effect in reaction time across all stimulation montages, which essentially reflects the cost of reorienting attention (Corbetta et al., 2008; Vossel et al., 2006) and has been repeatedly demonstrated by prior work utilizing the Posner cueing task (Arif, Spooner, Wiesman, Embury et al., 2020; Arif, Wiesman et al., 2020; Proskovec et al., 2018; Spooner, Wiesman, Proskovec, Heinrichs‐Graham, & Wilson, 2020).

Our most important findings were likely the decreased neural validity effects seen in occipital theta and occipital and parietal alpha responses following cathodal stimulation. In regard to the theta differences, reduced occipital theta oscillations during reorientation were seen following cathodal relative to both anodal and sham conditions. Such theta responses have been intimately tied to the initial encoding and sampling of the target stimulus (Busch, Dubois, & Van Rullen, 2009; Fries, Reynolds, Rorie, & Desimone, 2001) and been shown to be comparatively stronger during invalid targets (Proskovec et al., 2018). Particularly in the context of reallocation of attention, a previous transcranial magnetic stimulation (TMS) study showed that theta stimulation in retinotopic areas modulated attentional reorientation (Dugué, Roberts, & Carrasco, 2016), and the similar findings were reported by a later study, which employed a psychophysical task to manipulate attentional reorienting (Senoussi, Moreland, Busch, & Dugué, 2019). Taken together, our findings of increased theta power during valid compared to invalid trials following cathodal stimulation and a reversed pattern following sham and anodal stimulation fits well with the notion of an anodal‐excitation and cathodal‐inhibition dichotomy (Nitsche & Paulus, 2000, 2001), as mentioned in the introduction.

In contrast to occipital theta oscillations, alpha oscillatory responses in the occipital cortices generally reflect a decrease in local power (i.e., a desynchronization). Thus, the positive alpha validity effect in the cathodal condition also reflects a stronger response in the left occipital and parietal cortices during valid compared to invalid trials, while the opposite was observed in the anodal condition. Stronger alpha oscillations (i.e., increased desynchronization) have been extensively studied and it is generally believed that such responses reflect the disinhibition of local neural processing and thus active cortical engagement (Handel, Haarmeier, & Jensen, 2011; Michels et al., 2010; Murta, Leite, Carmichael, Figueiredo, & Lemieux, 2015; Spaak, de Lange, & Jensen, 2014). Thus, this finding may suggest stronger disinhibition of local neural populations following anodal stimulation, primarily in occipital cortices, to allow additional processing of incoming visual stimuli and the temporal shift during invalid trials. Interestingly, the same directionality of the alpha neural responses following anodal and cathodal stimulation were observed in the left parietal and these potentially also relate to the greater disinhibition and increased processing. These alpha oscillations in the parietal cortex were not surprising, as numerous previous reports have emphasized its role in attentional reorientation. More specifically, the phenomena of attentional disengagement from an expected target site, which is an essential subcomponent of attentional reorientation, has been shown to be more susceptible to the loss of parietal functioning by lesion studies (Posner, Walker, Friedrich, & Rafal, 1984; Posner, Walker, Friedrich, & Rafal, 1987). Additional evidence comes from neuroimaging studies targeting the parietal cortex with tDCS (Minamoto et al., 2014) and TMS (Capotosto, Corbetta, Romani, & Babiloni, 2012). Like theta, stronger alpha oscillations to invalid versus valid trials following anodal stimulation may imply greater neuronal deployment and thus systematic parietal engagement to meet augmented demands of the task during invalid trials. Interestingly, following anodal stimulation of the occipital cortices, we also observed specific coupling between our neural (i.e., parietal alpha validity) and behavioral indices (i.e., reaction time), such that smaller alpha validity effects in the parietal cortices were associated with faster responses on the task. This finding may indicate that the neural processing cost of attention reorientation was smallest following anodal stimulation, which could reflect the greater disinhibition of neural processing alluded to above following anodal occipital stimulation and such would be consistent with the notion of anodal‐excitation and more broadly polarity‐specific effects on the concentration levels of inhibitory neurotransmitters (Clark, Coffman, Trumbo, & Gasparovic, 2011; Heimrath et al., 2020; Krause, Márquez‐Ruiz, & Cohen Kadosh, 2013).

Finally, besides the stimulation by task condition interaction effects observed for theta and alpha oscillations, another major finding was the main effect of stimulation montage on right (and marginally left) visual gamma activity in occipital cortices. In the light of extant literature, the stronger gamma responses following anodal compared to cathodal stimulation is a very interesting finding and in accordance with a prior MEG study that investigated the impact of offline occipital tDCS on the gamma oscillations that are known to be induced by spatial grating stimuli, as well as a latter study that found that occipital cathodal stimulation reduced local spontaneous gamma activity (Wilson et al., 2018). Though not quantified in the present study, the observed impact of anodal stimulation on gamma oscillatory activity might be secondary to altered local GABAergic processing based on the findings of Kujala et al. (2015), who found negative coupling between GABA_A_ receptor density in the primary visual cortex and gamma power during a visual task in a multi‐modal MEG and Flumazenil‐PET study (Wilson et al., 2018). Consistent with this view, several GABA magnetic resonance spectroscopy studies have shown that anodal stimulation modulates local GABA concentration in visual and motor cortices (Bachtiar et al., 2018; Bachtiar, Near, Johansen‐Berg, & Stagg, 2015; Kim, Stephenson, Morris, & Jackson, 2014; Patel et al., 2019; Stagg et al., 2009; Stagg, Bachtiar, & Johansen‐Berg, 2011). Although some caution is warranted, as two previous MEG studies that employed a slightly different tDCS montage (i.e., Oz‐Cz) than the current study reported no effect of anodal stimulation on occipital gamma oscillations during visual processing (Hanley, Singh, & McGonigle, 2016; Marshall et al., 2016). However, this discrepancy might be explained by the differences in experimental methods, such as the use of conventional tDCS, concurrent tDCS with MEG (i.e., online), and smaller sample sizes in these studies as opposed to the current work.

Before closing, it is important to acknowledge the limitations of this study. Most importantly, though our neural findings are intriguing, owing to the lack of a concise understanding of the mechanism of action of tDCS, some of our interpretations remain speculative. Second, our alpha findings should be interpreted with caution. Though we observed polarity‐based differences in the power of alpha oscillations, neither of the active stimulation conditions differed significantly from sham, which limits interpretation. Further, we focused on a narrow parameter range of tDCS, mainly 20 min of 2.0 mA stimulation. Future work should examine a broader‐ parameter range, including longer and stronger stimulation. Along the same lines, we focused on occipital HD‐tDCS and cannot comment on the impact of HD‐tDCS on other brain regions serving attentional reorientation. Despite these limitations, the current study provides novel information regarding the neurophysiological impact of offline occipital HD‐tDCS on attentional reorientation, and our findings highlight spectrally‐specific effects that depend on the polarity of the stimulation, and further suggest an important framework for future studies to probe the GABA‐gamma link with tDCS. Additionally, future work should evaluate whether the multispectral, polarity‐specific changes identified in this and related studies reflect changes in local cross‐frequency coupling of neural activity.

## CONFLICT OF INTERESTS

The authors declare that there are no conflict of interests.

## Data Availability

The data that support the findings of this study are available from the corresponding author upon reasonable request. All data will also be released from the NIMH Data Archive upon completion of the study.
